# Inter-rater reliability of muscle ultrasonography performed by multidisciplinary novice sonographers in the evaluation of critically ill patients with acute kidney injury requiring continuous kidney replacement therapy

**DOI:** 10.1080/0886022X.2025.2472990

**Published:** 2025-03-11

**Authors:** Felipe González-Seguel, Vinh Q. Tran, Chaitanya Anil Pal, Zan T. Shareef, Hayley P. Israel, Arimitsu Horikawa-Strakovsky, Yuan Wen, Benjamin R. Griffin, Javier A. Neyra, J. Pedro Teixeira, Kirby P. Mayer

**Affiliations:** a Department of Physical Therapy, College of Health Sciences, University of Kentucky, Lexington, KY, USA; b Center for Muscle Biology, University of Kentucky, Lexington, KY, USA; c Division of Physical Therapy, Department of Orthopaedics, University of New Mexico School of Medicine, Albuquerque, NM, USA; d Division of Nephrology, University of New Mexico School of Medicine, Albuquerque, NM, USA; e Division of Pulmonary, Critical Care, and Sleep Medicine, University of New Mexico School of Medicine, Albuquerque, NM, USA; f Math, Science, and Technology Center Program, Paul Laurence Dunbar High School, Lexington, KY, USA; g Institute for Biomedical Informatics, University of Kentucky, Lexington, KY, USA; h Department of Physiology, College of Medicine, University of Kentucky, Lexington, KY, USA; i Division of Nephrology, University of Iowa Hospitals and Clinics, Iowa City, IA, USA; j Department of Internal Medicine, Division of Nephrology, University of Alabama at Birmingham, Birmingham, AL, USA

**Keywords:** Acute kidney injury, critical illness, continuous renal replacement therapy, ICU-acquired weakness, muscle ultrasound, ultrasound training

## Abstract

Early diagnosis of muscle wasting in critically ill patients with acute kidney injury requiring continuous kidney replacement therapy (AKI-CKRT) may improve outcomes *via* timely rehabilitation and nutrition. Muscle ultrasound (MUS) has recently gained traction for assessing muscle atrophy in the intensive care unit (ICU) but requires training to achieve reproducibility. We evaluated the inter-rater reliability of MUS in patients with AKI-CKRT performed by multidisciplinary raters including nephrologists. Two blinded independent raters used portable ultrasound to acquire images of the rectus femoris (RF). All raters were clinicians routinely caring for patients with CKRT in the ICU and were initially novices in MUS. They underwent three two-hour teleconference training sessions in MUS led by an experienced physiotherapist. Inter-rater reliability was evaluated with intraclass correlation coefficients (ICCs) [95% confidence interval] using a two-way random-effects model. We analyzed 54 MUS images (27 pairs) from nine patients at baseline (*n* = 16), day 3 (*n* = 6), day 7 (*n* = 8), ICU discharge (*n* = 10), hospital discharge (*n* = 10), and 1–3 months after discharge (*n* = 4). The mean (±standard deviation) values of RF thickness, cross-sectional area, and echointensity were 1.7 ± 1.4 cm, 4.6 ± 2.7 cm^2^, and 84.0 ± 17.7 AU, respectively. Reliability was excellent for RF thickness (ICC = 0.96 [0.91–0.98], *p* < 0.001) and cross-sectional area (ICC = 0.92 [0.83–0.96], *p* < 0.001) but poor for echointensity (ICC = 0.41 [0.04–0.68], *p* < 0.05). These results demonstrate reliable assessment of muscle size in patients with AKI-CKRT using ultrasound performed by multidisciplinary novice sonographers trained *via* teleconference, suggesting that this methodology may be useful in future studies of muscle wasting in patients with AKI-CKRT.

## Abstract (plain language summary)

Trial Registration: NCT05287204

## Introduction

Skeletal muscle atrophy is a common consequence of critical illness which develops rapidly in the intensive care unit (ICU) and is associated with long-term mortality, impaired functional status, and decreased quality of life [1]. For example, a recent meta-analysis of over 50 studies and over 3,000 critically ill patients found an overall prevalence of ICU-acquired weakness of 48% with an average rate of loss of muscle of nearly 2% per day during the first week of ICU admission [1].

Both preclinical data and human studies suggest that muscle wasting in the ICU may be exacerbated by acute kidney injury (AKI), especially AKI requiring kidney replacement therapy (KRT). AKI appears to trigger, through a variety of inflammatory mediators and metabolic pathways, enhanced muscle catabolism, which is exacerbated in patients requiring KRT by the nonselective clearance of amino acids and small peptides [2]. Continuous KRT (CKRT) in particular may contribute to the development of muscle wasting, with recent studies demonstrating that CKRT can remove up to 10–20 g of amino acids per day [3,4].

The timely diagnosis of muscle wasting during critical illness may improve patient outcomes by allowing for prompt delivery of targeted rehabilitation and enhanced nutritional support. Ultrasonography is a noninvasive, low-cost, and radiation-free examination tool that has gained significant traction for assessing muscle mass and architecture in the ICU [1]. However, a primary limitation of muscle ultrasonography is the training required to reproducibly acquire images [5]. Several studies have demonstrated that muscle ultrasound on ICU patients has high inter-rater reliability when performed by expert sonographers (mostly physical therapists), with intraclass correlation coefficients (ICCs) ranging between 0.70 and 1.00 [6,7]. However, the inter-rater reliability of muscle ultrasound performed by healthcare providers routinely caring for patients with AKI requiring KRT in the ICU is unknown [8,9]. We aimed to evaluate the inter-rater reliability of the use of ultrasonography to measure muscle wasting performed by such multidisciplinary healthcare providers without prior expertise in muscle ultrasonography trained *via* a teleconference format.

## Methods

We performed a preplanned reliability analysis of the data generated at one site, University of New Mexico Hospital (UNMH), participating in a multicenter prospective observational study which evaluated the rate of muscle wasting in critically ill patients with AKI requiring CKRT. The detailed protocol was registered on clinicaltrials.gov (NCT05287204) on March 10, 2022; was approved by the University of Kentucky Office of Research Integrity Medical IRB, which served as the single IRB for this multisite study (IRB #71153; initial approval June 7, 2022; protocol version 2 dated approved January, 7 2023); and has been previously published [10]. All subjects provided informed consent and study procedures were carried out in accordance with the Declaration of Helsinki.

The CKRT program at UNMH utilizes PrismaSol and PrismaSATE solutions delivered by Prismaflex or PrisMax devices with M100 or HF1000 hemofilters (all from Baxter International, Deerfield, IL) and a standardized CKRT prescription of continuous venovenous hemodiafiltration at an initial total effluent dose of approximately 25 mL/kg/h with 1-to-1 ratio of replacement fluid and dialysate, with dose adjustment thereafter as needed based on serial laboratory monitoring. Approximately 250 patients are treated with CKRT over approximately 30,000 therapy hours each year at UNMH [11].

All of the sonographers at UNMH had varying degrees of prior experience with ultrasound in general, but all five were novices in musculoskeletal ultrasound prior to the study. Therefore, they each underwent three two-hour sessions of ultrasound training *via* teleconference led remotely by a physiotherapist (KPM) with >8 years of ultrasound experience [7]. The training involved instruction and practice performing a minimal-to-no probe compression technique using excess ultrasound gel. Additionally, the investigators were instructed to practice a minimum of ten image acquisitions from healthy individuals before study initiation.

During the study, two blinded raters at the site used portable ultrasound devices to independently acquire three images of rectus femoris at baseline (within 48 h after CKRT initiation), day 3, day 7, ICU discharge, hospital discharge, and 1–3 months after discharge. Rater 1 at each time point was either a physical therapist (VQT), an intensivist (HPI), or a nephrology fellow (ZTS or CAP), while rater 2 each time point was the site primary investigator, a nephrologist-intensivist (JPT). In other words, at each time point, images generated by JPT were compared to images generated by one of the other four local coinvestigators (CAP, HPI, VQT or ZTS). Images at each time point were obtained within four hours of each other using a Butterfly iQ handheld ultrasound device (Butterfly Network Inc., Burlington, MA) with a linear probe (5–15 Hz), auto-gain setting, and a depth of 6 cm.

The acquired images were analyzed by three coinvestigators (AHS, YW and KPM) and the best of the three images obtained by each rater at each time point was selected to determine rectus femoris (1) muscle thickness, (2) cross-sectional area and (3) echointensity. Echointensity represents a measure of muscle quality and was quantified using grayscale histogram analysis as previously described [6]. Image analysis was carried out using MyoVision-US, a newly developed automated software tool utilizing a deep-learning artificial intelligence model [12]. The experienced physiotherapist (KPM) adjudicated measurements from MyoVision-US as needed.

The inter-rater reliability of the paired within-patient ultrasound measurements was quantified with ICCs generated using a two-way random-effects model. Intraclass correlation—a statistical methodology used commonly in studies of the reliability of ultrasound—quantitatively assesses the reliability of ratings by comparing the variability of different ratings of the same subject (in this case, the same patient at the same time point) to the total variation across all ratings and all subjects [6,7,13]. The *p*-value for an ICC is the probability of observing that ICC if no correlation existed (e.g. the null hypothesis is that ICC = 0), and a *p*-value <0.05 represents a statistically meaningful level of agreement. The ICC values represent the strength of this agreement on a scale from 0 to 1, with 0.75–1.0 considered good-to-excellent reliability, 0.5–0.75 considered moderate, and 0–0.5 considered poor [14]. Based on previous ultrasonography literature, ICCs >0.75 for novice sonographers are considered meaningful [6,7]. The absolute agreements between measurements were visualized using Bland-Altman plots.

## Results

**Figure 1.**
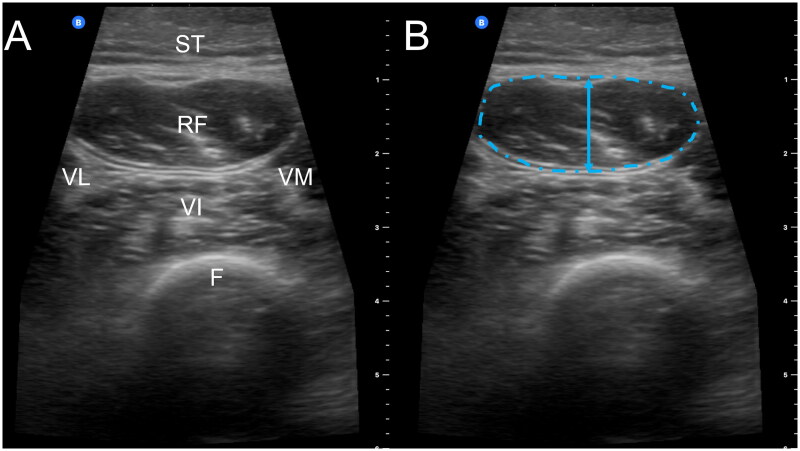
(A) Ultrasound image of the quadriceps acquired at two-thirds the distance from the anterior superior iliac spine to the superior aspect of the patella with 6 cm of depth demonstrating subcutaneous tissue (ST), rectus femoris (RF), vastus lateralis (VL), vastus medialis (VM), vastus intermedius (VI), and femur (F). (B) The blue arrow denotes rectus femoris muscle thickness while the blue dashed outline delineates the rectus femoris cross-sectional area. Rectus femoris echointensity is calculated from the pixel density within the rectus femoris cross-sectional area.

Two independent raters acquired a total of 162 ultrasound images from nine adults requiring CKRT. One of three repeated ultrasound images from each rater at each time point was evaluated. Ultimately, 54 ultrasound images (27 pairs) were included in the reliability analysis from the following time points: baseline (*n* = 16), day 3 (*n* = 6), day 7 (*n* = 8), ICU discharge (*n* = 10), hospital discharge (*n* = 10), and 1–3 months after discharge (*n* = 4). A representative ultrasound image is shown in Figure 1.

Baseline clinical characteristics (median [IQR]) of the cohort include age 57 [34–62] years, BMI 30 [26–47] kg/m^2^, and Charlson Comorbidity Index 4 [2–6]. Four subjects (44%) were women, and four (44%) were mechanically ventilated. Median hospital length of stay was 13 [10–25] days, and median duration of CKRT was 2 [1–6] days. Three (33%) subjects died before hospital discharge.

**Figure 2.**
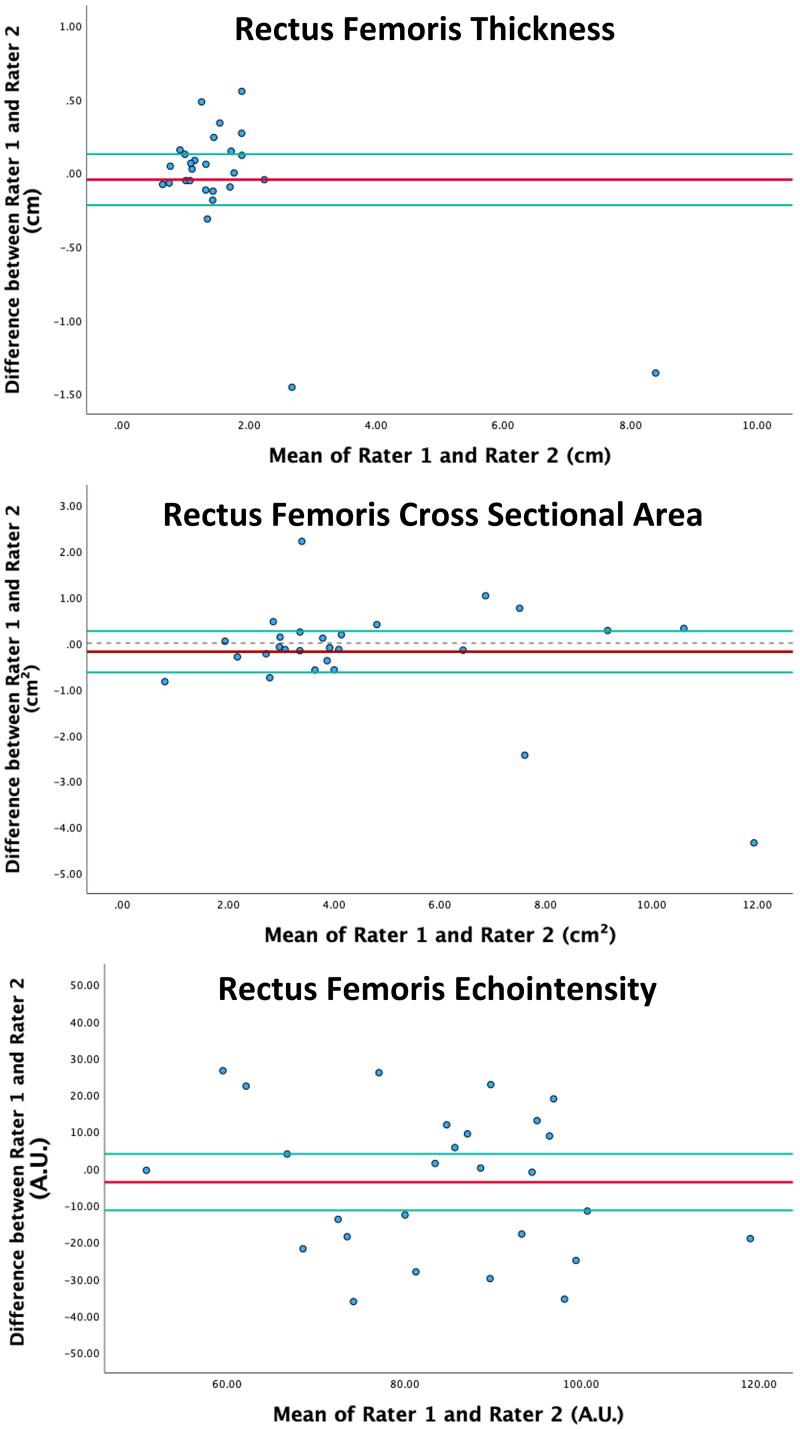
Bland-Altman plots for absolute agreement between rater 1 and rater 2 across muscle ultrasound parameters. X-axis represents the mean values of all raters. Y-axis represents the difference values between rater 1 and rater 2. The red lines represent the mean differences, and the green lines represent upper and lower limits of agreement around the mean differences. In each figure the number of blue circles is 27, representing the total number of pairs of images from the two raters included in the analysis.

The mean (± standard deviation) values of rectus femoris thickness, cross-sectional area, and echointensity were 1.7 ± 1.4 cm, 4.6 ± 2.7 cm^2^, and 84.0 ± 17.7 A.U., respectively. The results of the inter-rater reliability analysis are presented in Table 1. The ICCs were 0.96 for muscle thickness, 0.92 for cross-sectional area, and 0.41 for echointensity. Bland-Altman plots displaying the absolute agreement between measurements for all three ultrasound parameters for all 27 time points are shown in Figure 2.

**Table 1.** Inter-rater reliability of rectus femoris ultrasound parameters obtained from critically ill patients with acute kidney injury requiring kidney replacement therapy.

	Rectus femoris thickness	Rectus femoris cross-sectional area	Rectus femoris echointensity
Intraclass correlation coefficient	0.96	0.92	0.41
95% confidence interval	0.91–0.98	0.83–0.96	0.04–0.68
*P*-value	<0.001	<0.001	0.016

## Discussion

Our analysis suggests that multidisciplinary healthcare providers who were previously inexperienced in muscle ultrasonography—namely nephrologists, intensivists, and physiotherapists—can be trained using a teleconference format to achieve excellent reliability in the ultrasonographic assessment of the size of skeletal muscle of critically ill patients with AKI requiring CKRT. This degree of reliability is comparable to prior studies analyzing muscle ultrasound in other ICU subpopulations [6,7], but extends the findings to nephrologists as sonographers. Collectively, our results and these prior studies suggest that the entire multidisciplinary team caring for patients with AKI requiring KRT in the ICU can be quickly trained to reliably assess changes in muscle size using ultrasound.

While the multidisciplinary providers in our study obtained excellent reliability in muscle size, the reliability was only poor for echointensity [15]. This discrepancy may be explained by novice raters failing to make necessary small adjustments in probe angle/tilting [5]. Additionally, variable levels of adiposity or edema, which is common in patients with AKI, may cause soundwave distortion and contribute to imprecise readings [8,16].

Strengths and novel aspects of this study include the multidisciplinary team who underwent remote training to perform muscle ultrasound. In addition, this is one of the first studies reporting reliability of muscle ultrasound findings in this critically ill subpopulation with AKI receiving CKRT at high risk of muscle atrophy [2]. Furthermore, rather than using particularly expensive or sophisticated ultrasound equipment, we achieved these results using a handheld device considered to have moderate image quality [17], suggesting our results are likely to generalize to other widely available portable ultrasound devices.

Limitations of our analysis include the single-center nature, which may limit the generalizability of our results, and the small sample size, which reduces the confidence in our findings. These results will need to be reproduced in larger multicenter studies. However, notably the sample size of interest in this sub-study is not the number of patients, but rather the number of images, and with >50 images analyzed, the rater-to-image ratio achieved was well within the recommended range [13] to estimate precise ICCs, as reflected in the 95% confidence intervals for our ICCs (Table 1). Furthermore, our study relied on a single modality of assessment of skeletal muscle, ultrasonography, and, in a better-resourced study, comparisons with other methods of muscle evaluation—such as computed tomography (CT), bioimpedance analysis (BIA), electromyography (EMG), and muscle biopsy—would have been informative. However, given the cost and radiation associated with CT, studies of critical illness myopathy using CT typically consist of retrospective studies of scans obtained for clinical indications, whereas the currently available data to support BIA in the diagnosis of critical illness myopathy are limited and mixed at best [18–22]. Likewise, while EMG and biopsy are considered gold standards for the diagnosis of critical illness myopathy, they are both invasive and expensive tests rarely performed even for research [1,2]. For all these reasons, ultrasonography is now overwhelmingly the most commonly employed method to assess muscle mass in research studies of muscle wasting in the ICU [1]. Additionally, though we initially planned for in-person training of all investigators, due to the COVID-19 pandemic we resorted to a teleconference format. Online training may have reduced hands-on feedback to the learners, potentially contributing to errors in precise manipulation of ultrasound probe angle that may have resulted in imprecision in the assessment of echointensity. However, our findings overall suggest that this teleconference format would be reproducible and potentially scalable to larger multicenter trials focused on the evaluation of muscle size. Moreover, though edema may potentially contribute to decreased reliability of the echointensity measurements, our protocol did not include collection of data on volume status. Finally, we were also unable to report a variety of other potentially relevant patient characteristics that could influence the development of muscle wasting, such as treatment with paralytic agents or corticosteroids. However, given this reliability analysis is comparing paired images obtained from the same patient at the same time point from two different raters, the factors influencing the development of muscle wasting in a given patient would not necessarily be expected to influence these within-patient comparisons.

In conclusion, our results demonstrate the reliable use of ultrasonography by a multidisciplinary group of healthcare providers—including nephrologists—in the assessment of muscle size in critically ill patients with AKI requiring CKRT. This reliability was achieved *via* teleconference training despite the fact that all five sonographers were previously novices in muscle ultrasonography. While point-of-care ultrasound by nephrologists is rapidly gaining popularity and is being increasingly integrated into nephrology fellowship training programs and nephrology conference courses, training curricula for nephrologists typically do not emphasize musculoskeletal ultrasound [23]. Though additional larger multicenter studies are needed to confirm these findings, our results suggest that the methodology of teleconference ultrasound training can be applied to broader studies carried out by nephrologists, intensivists, and physiotherapists on the impact of acute or chronic kidney failure and KRT on muscle wasting. However, our findings also reinforce the need for additional research on how to optimize ultrasound training for muscle echointensity measurements (i.e. muscle quality assessments) in acutely ill populations.

## Data Availability

The data presented in this manuscript are available upon reasonable request, pending regulatory approval by the institutional review boards of the University of New Mexico (data source) and the University of Kentucky (single IRB for this multicenter study).

## Prior publication

A summary of the data presented in this manuscript were previously presented as an oral abstract at the American Society of Nephrology Kidney Week in October 2024 in San Diego, CA, and subsequently published as a printed abstract: Teixeira JP, Gonzalez-Seguel F, Tran V, Pal CA, Shareef Z, Israel H, Horikawa-Strakovsky A, Wen Y, Griffin BR, Mayer K. (2024). Multidisciplinary Inter-rater Reliability of Muscle Ultrasonography in Patients with AKI Requiring Continuous Kidney Replacement Therapy: TH-OR17. *Journal of the American Society of Nephrology*, 35(10S), 10-1681. doi: 10.1681/ASN.20246ggwcv61.
